# Uncovering the Moderating Role of Grit and Gender in the Association between Teacher Autonomy Support and Social Competence among Chinese Undergraduate Students

**DOI:** 10.3390/ijerph17176398

**Published:** 2020-09-02

**Authors:** Yongfeng Ma, Chunhua Ma, Xiaoyu Lan

**Affiliations:** 1 College of Educational Science and Technology, Northwest Minzu University, Lanzhou 730030, China; mayongfeng@xbmu.edu.cn (Y.M.); mch@xbmu.edu.cn (C.M.); 2 Department of Developmental Psychology and Socialization, University of Padova, Via Venezia, 8, 35131 Padova, Italy

**Keywords:** teacher autonomy support, social competence, grit, gender, undergraduate student, Chinese

## Abstract

Does teacher autonomy support significantly facilitate the social competence of undergraduate students in a collective cultural context? Does this study association vary by individual characteristics, such as grit and students’ gender? To answer these research questions, we examine the association between teacher autonomy support and social competence. Moreover, we ascertain whether two dimensions of grit (perseverance and consistency) and/or gender may moderate this association. A convenience sample of 1009 Chinese undergraduate students (*M*_age_ = 20.66; *SD* = 1.30, 47.4% female) was involved in this study, and they were asked to complete a set of self-report questionnaires online. Results of linear regression analyses revealed that (a) teacher autonomy support was positively associated with social competence, and (b) when reporting higher levels of consistency, this association was significantly positive for both males and females; by contrast, when reporting lower levels of consistency, this association was only significant for males but not for females. The current study indicates the beneficial role of teacher autonomy support in undergraduate students’ social competence in a collective cultural context. Furthermore, university-based intervention or prevention programs should focus on facilitating teacher autonomy support for all students; it is noteworthy that, for female students, enhancing consistency should also be incorporated into these programs.

## 1. Introduction

Social competence is defined as the ability to engage in satisfying social interactions aimed at initiating, facilitating, and maintaining successful relationships [1,2]. Previous studies have well documented that subjective appraisal of social competence is linked to a broad range of adaptive psychosocial functioning, such as better psychological wellbeing, high prosocial tendencies, and less depressive symptoms [2,3,4]. Given the importance of social competence in facilitating optimal psychosocial adjustment among individuals, it would be valuable and meaningful to further address its correlates. Surprisingly, much of the research so far has focused on children and adolescents in the precollege context [5]; by contrast, relatively little research attention has been paid to undergraduate students.

In terms of undergraduate students, they face several difficult challenges, such as significant academic stress and possible job-seeking competition [6,7,8]. Indeed, moving to a residential college and being physically separated from their family support system, undergraduate students have to assemble and maintain a supportive social network. The social ties established may help undergraduate students become more socially integrated, which is crucial to their successful adaption in this period [4]. This is particularly true in Chinese society. With rapid economic development, Chinese undergraduate students encounter salient academic challenges and increasingly fierce competition [6,8], in which cultivating undergraduate students’ various abilities (e.g., social skills) is essential. Moreover, with its embedded Confucianism, the Chinese culture attaches significant importance to social harmony and interpersonal interactions [9,10]. Therefore, further investigation into the correlates of social competence among Chinese undergraduate students would be particularly meaningful in informing college-based intervention or prevention programs.

To investigate the correlates of social competence, we refer to the socioecological framework [11]. This is because social competence is an ecological phenomenon, which has been developed over time as a consequence of multiple interactions across distinct contexts [12,13]. Hence, this framework would provide a comprehensive understanding of the contextual and individual factors associated with social competence. To be specific, according to this framework, individuals are embedded in various layers of the system, and their development occurs in multiple interactions between situations (e.g., teachers) and persons (e.g., grit and gender) [14,15]. Therefore, it is essential to examine how these factors directly and interactively contribute to social competence.

To briefly summarize, we aim to investigate the association between teacher autonomy support and social competence and further unravel whether grit and gender may moderate this study association in a large sample of Chinese undergraduate students. In the following sections, we conduct the literature review per each study variable, starting from the presentation of teacher autonomy support.

### 1.1. Teacher Autonomy Support

Autonomy support refers to an interpersonal style in which significant figures (e.g., teachers) take their students’ perspective into account, present rationales for demands, acknowledge their feelings, and provide opportunities for choice and self-determination [16,17,18]. In this study, we center on teacher autonomy support based on the following considerations. First, undergraduate students spend most of their time in college, and the salient role of the teacher–student relationship in this context makes it more available to intervene in. Second, influenced by Confucian culture heritage, respecting teachers at school and emphasizing higher education achievement are essential socialization goals for Chinese individuals. In this context, teachers are considered as the propagators of human knowledge, the developers of student intelligence, and the counselors of youth [19]. A teacher’s work is regarded as a “candle” to light up students’ future development, significantly influencing their social behaviors and values. Despite these salient features, most of the extant research has studied children and adolescents in the precollege context, but relatively few research efforts have been devoted to undergraduate students.

According to the self-determination theory (SDT), autonomy is one of the individual’s basic psychological needs [16]. SDT asserts that teachers can support students’ psychological needs in the school environment, which plays a vital role in facilitating their inner motivation [20,21]. It is well documented that teacher autonomy support plays an important role in the adaptive functioning of individuals [22], also including peer relatedness and competence [20]. This is mainly because the autonomy-supportive motivating style in the classroom catalyzes engagement-fostering motivations and encourages students to independently explore and validate their perspectives, which may engender a sense of control and mastery and facilitate their optimal development [18]. Despite these theoretical and empirical indications, there are still some open questions that merit further investigation. For instance, the beneficial role of teacher autonomy support in social competence is still not clear-cut in a collectivistic cultural context. Some researchers argue that the positive role of autonomy support on individuals’ adaption in a collective setting may not be salient, as conformity and interdependence are highly emphasized in collectivistic cultural values [23].

Furthermore, as documented by prior research and our theoretical framework [11,14], student adaptive functioning is not only related to contextual variables, such as teacher autonomy support, but also linked to individual factors, such as grit.

### 1.2. Grit

Grit refers to perseverance and passion in achieving a long-term goal [24,25]. Although grit is regarded as a relatively stable trait [24,26], empirical studies also indicate that grit is malleable [27,28,29]. Thus, documenting the role of grit in the association between teacher autonomy support and the social competence of undergraduate students is particularly valuable, as this finding may provide some possible insights into designing intervention or prevention studies. According to the extant literature, grit is positively linked to success within a broad range of academic settings [30,31], which has also been extended to college students [32]. Despite such research progress, little attention has been paid to the possible linkage between grit and outcomes beyond academic settings.

Albeit limited, a burgeoning body of research has indicated that gritty individuals often exhibit better adaptive psychosocial functions, such as psychological wellbeing [33,34,35], prosocial behavior [28], personal relationships [10], and less mental distress [36]. Indeed, grit is a future-oriented motivation and might induce a sense of hope and determination, which further improves a sense of meaning in individuals’ lives and facilitates their positive interpersonal interactions. This is particularly valued in Chinese culture, as Confucianism puts a great emphasis on perseverance and diligence [28,37].

Moreover, according to recent empirical studies, the moderating role of grit between contextual variables (e.g., teacher autonomy support) and adjustment outcomes has been illustrated, which gives us some indications of possible moderation direction of grit in the current study. For example, Lan and his colleagues (2019) have found that the positive association between parental autonomy support and prosocial behavior is significantly heightened for Chinese youths reporting higher levels of the overall score of grit (as opposed to lower levels of grit) [28]. Although these findings are of great importance, it prohibits a comprehensive understanding of the role of different dimensions of grit in psychosocial outcomes.

Grit consists of two facets: perseverance of effort and consistency of interests [24,25]. To be specific, perseverance refers to sustaining personal effort and determination to accomplish a goal, and consistency values commitment to interests that may lead to goal achievement. An emerging body of research has noted that these two facets may reflect independent constructs, contributing to individuals’ adaptive functions distinctively [33,38]. Moreover, a meta-analytic review has shown that perseverance has significantly stronger criterion validity than consistency in academic performance [30]. In a similar vein, in the collective cultural setting, perseverance has been documented to be more salient than consistency in predicting key psychological outcomes such as academic engagement and subjective wellbeing [33]. Despite these pieces of evidence, it is still underexplored whether perseverance and consistency may distinctively interact with teacher autonomy support, contributing to social competence among undergraduate students.

Furthermore, it is noteworthy that there is conflicting evidence about the presence of gender differences in grit. Some findings reveal no significant differences in gender [25,35], also in a sample of university students [39]. However, other empirical studies show that females exhibit higher levels of grit than males [40,41]. Surprisingly, little research attention has been paid to the possible gender differences in grit in a collective cultural context. Given the conflicting evidence in the extant literature and distinctive socialization goals for different genders in Chinese society, we further investigate whether grit may interact with individuals’ gender to explain the variance in the linkage between teacher autonomy support and social competence.

### 1.3. Gender Differences

It is well-documented that females usually outperform males in academic engagement and performance, influencing their teacher–student relationship distinctively [42]. Influenced by traditional Chinese culture, females are generally considered sensible and more likely to solve interpersonal misunderstandings and maintain harmonious relationships than males. Therefore, it is conceivable that the beneficial effect of teacher autonomy support on social competence may be stronger for females than males.

Furthermore, in traditional Chinese culture, individuals generally adhere to inequitable gender norms. For example, males are expected to be more psychologically firm and emotionally independent than females [43]. As described by well-known Chinese proverbs, males are often portrayed as “big boys who rather bleed without tears” or to “take it like a man”. To maintain this gender norm, males are highly expected to persist with effort in the face of difficulties and to solve these challenges tenaciously. Due to these salient cultural values, we expect that students’ gender, along with grit, may moderate the association between teacher autonomy support and social competence.

### 1.4. The Present Study

**Figure 1:**
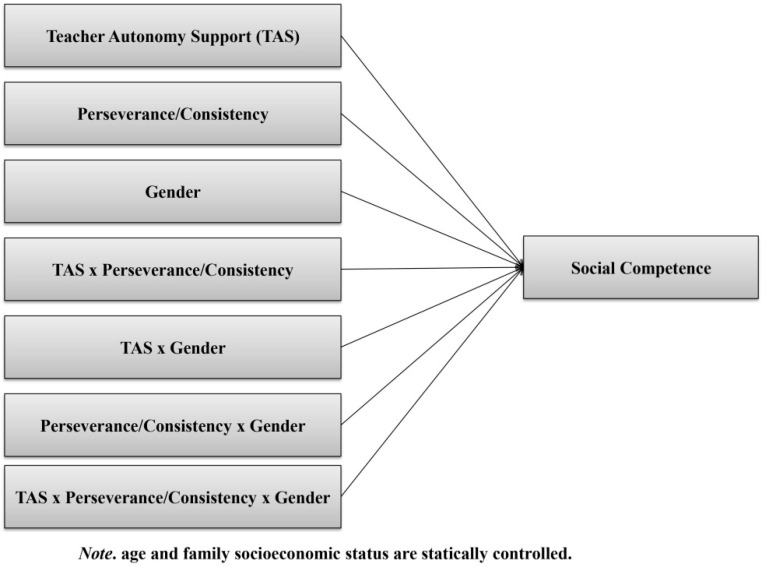
A hypothesized model of the direct and interactive associations of teacher autonomy support, two dimensions of grit, and students’ gender with social competence. Note: We conceptually regard teacher autonomy support as an independent variable and the two dimensions of grit and students’ gender as moderators. Although the interaction between two moderators (i.e., Perseverance/Consistency × Gender) on social competence is not our research focus, it should be established as one of the necessary steps to create a three-way interaction term.

The purpose of the present study is twofold: (a) We investigate the association between teacher autonomy support and social competence in a sample of Chinese undergraduate students, and (b) we examine whether the two facets of grit (i.e., perseverance and consistency) and/or gender may moderate this association. Furthermore, as indicated by previous studies [2,4,44], several sociodemographic variables (e.g., age and family socioeconomic status (SES)) are potentially linked to our dependent variable (i.e., social competence). Thus, we statistically control those variables in this study. A graphical representation of our hypothesized model is shown in Figure 1.

According to the socioecological framework and the relevant empirical studies reviewed above, we propose the following hypotheses (H):

**Hypotheses 1 (H1).**  *Teacher autonomy support is positively associated with social competence in Chinese undergraduate students;*

**Hypotheses 2a (H2a).**  *Grit may moderate the association between teacher autonomy support and social competence. To be specific, high levels of grit may strengthen the positive linkage between teacher autonomy support and social competence (vice versa, low levels of grit may weaken this positive association), and the facilitating role of grit may strongly rely on the perseverance dimension instead of consistency;*

**Hypotheses 2b (H2b).**  *Gender may also moderate these associations. Specifically, we anticipate that high levels of grit may strengthen the association between teacher autonomy support and social competence, which is more pronounced for males than females.*

## 2. Method

### 2.1. Participants and Procedures

Before conducting this investigation, this study was ethically approved by the Institutional Review Board affiliated with Northwest Minzu University. Through personal networks, the authors contacted several responsible school principals and professors in public universities located in Gansu Province and explained to them this survey’s purposes. Finally, three public universities agreed to participate in this investigation, and we relied on the convenience sampling method to recruit participants based on their availability and willingness to take part. After obtaining school authorities’ approvals, the authors shared a unique QR code with teachers and informed them to share this QR code with possible participants during public school hours. Participants were asked to scan this QR code and followed the online instructions to fill in the questionnaires. These questionnaires were selected based on well-documented psychometric properties and a proper number of items, potentially decreasing participants’ burden and improving the participation rate. At the beginning of this survey, we briefly stated our research purposes for the participants. Confidentiality and anonymity of this study were strictly guaranteed during the research process. Moreover, participation was voluntary, and participants could withdraw from this survey at any time.

Based on our hypothesized model (see Figure 1), we could insert nine predictors at most (teacher autonomy support, perseverance/consistency, students’ gender, age, family socioeconomic status, and possible two- and three-way interactions) in the linear regression model. Based on extant research on social competence [45], low-to-medium effect sizes (ƒ^2^ = 0.02–0.15) and 0.95 statistical power were deemed as desirable. Therefore, as decided by power analysis, the appropriate sample size of this study should range from 167 to approximately 1000. Keeping this in mind, we successfully recruited a large sample to address our study associations. A total of 1009 undergraduate students, aged from 18 to 25 years old, were involved in the current study. Their mean age was 20.66 years (*SD* = 1.30). Of these participants, 52.6% were males (*n* = 531) and 47.4% were females (*n* = 478). Moreover, 56.0% students (*n* = 565) were in their first year of college, 29.8% (*n* = 301) were in their second year, and 14.2% (*n* = 143) were in their third year. In addition, 65% of students belonged to the Han ethnic group (the majority ethnic group in China) [6] and 32.7% were ethnic minorities (2.3% were missing values). In terms of their parents’ educational background, most fathers (64.5%) and mothers (75.9%) had completed middle school or lower. With regard to their parents’ occupational status, most of their fathers and mothers were employees in companies or government agencies. Additionally, most students reported that their family monthly income was 3000–5000 RMB, which equals to 400–700 US dollars.

### 2.2. Measures

#### 2.2.1. Social Competence

Social competence was assessed using the Chinese translation of the social competence scale initially developed by Valkenburg and Peter (2008) [46]. This scale consists of 19 items and four dimensions, including the initiation of relationships (five items; e.g., “Start a conversation with someone you did not know very well?”), supportiveness (five items; e.g., “Listen carefully to someone who told you about a problem he or she is experiencing?”), the ability to self-disclose (five items; e.g., “Express your feelings to someone else?”), and assertiveness (four items; e.g., “Stand up for your rights when someone wrongs you?”). Each item was rated on a 5-point scale ranging from 1 (very difficult) to 5 (very easy). We used the mean score of all items to represent social competence in this study, with higher scores indicating higher social competence levels. Prior research has shown good reliability and validity of this scale [46,47]. In the present study, the internal consistency (measured by Cronbach’s alpha) of this scale was 0.89. Although Cronbach’s alpha is a commonly used indicator of reliability in social science, due to its limitations recognized by prior research [48,49], we also report McDonald’s omega of each scale in the present study. In the current sample, McDonald’s omega of this scale was 0.89.

#### 2.2.2. Teacher Autonomy Support

Teacher autonomy support was measured using the Chinese version of the Learning Climate Questionnaire (LCQ) [50]. This single-dimension scale consists of 15 items that are designed concerning specific learning settings, such as in college. Following the original guidelines of this scale, the wording of the items was slightly adapted, and the questions concerned the autonomy support of faculty members in general. Item examples are, “my professor tries to understand how I see things before suggesting a new way to do things”, “I feel that my professor provides me choices and options”. Participants were asked to assess each item on a 7-point Likert scale, ranging from 1 (strongly disagree) to 7 (strongly agree). The score of teacher autonomy support was calculated by averaging all items, with higher values indicating higher levels of perception of autonomy support from teachers. Previous research has demonstrated good internal consistency for this scale in college students [51] and also in Chinese youths [14]. In the present study, the Cronbach’s alpha of this scale was 0.94, and McDonald’s omega of this scale was 0.94.

#### 2.2.3. Grit

Grit was assessed using the Chinese version of the grit scale (8 items) [25]. This scale has been previously validated in Chinese youths, showing adequate reliability and validity [52]. Of this scale, four items refer to the perseverance of effort (e.g., “I am diligent”, “Setbacks do not discourage me. I do not give up easily”), and four reverse-coded items involve the consistency of interests (e.g., “I often set a goal but later choose to pursue a different one”, “New ideas and projects sometimes distract me from previous ones”). Participants were instructed to rate each item on a scale ranging from 1 (not at all like me) to 5 (very much like me). All items per each dimension were averaged to obtain the scores of perseverance and consistency, with higher scores indicating higher levels of perseverance and consistency, respectively. Prior research has exhibited good internal consistency of this scale in Chinese college students [26]. In this study, Cronbach’s alphas were 0.70 and 0.67 for perseverance and consistency (McDonald’s omegas were 0.74 and 0.69 for perseverance and consistency).

#### 2.2.4. Sociodemographic Characteristics

We asked participants to report their age, gender, grade level, ethnicity, parental educational background, parental occupational status, and family monthly income. Following prior research [14,44], we operationalized family SES by parental educational background, parental occupational status, and family monthly income. The scores of these three variables were standardized first and then summarized into a composite score representing family SES, with higher scores indicating higher levels of family SES.

### 2.3. Data Analysis

We used SPSS 21.0 [53], Jamovi 1.1.9.0 [54], and R software [55] to perform data analysis. First, we computed means, standard deviations, and zero-order correlations of study variables. To label the strength of the correlation coefficients, we regarded the absolute value of *r* < 0.35 as weak correlations, 0.36–0.67 as moderate, and >0.68 as strong [56]. Second, we conducted a linear regression analysis using Jamovi to examine the direct and interactive associations of teacher autonomy support, grit, and students’ gender with social competence. In this step of the analysis, the categorical variable (i.e., gender) was dummy-coded, and continuous variables were centered before being entered into the regression model to avoid overestimations of parameters [57].

Before performing the linear regression analysis, the assumptions of linear regression were examined by relevant graphs and pertinent analyses. To be specific, we relied on the values of skewness and kurtosis to test univariate normality and the quantile-quantile (Q-Q) plot to detect multivariate normality of study variables. Apart from the Q-Q plot, we also calculated Mardia’s multivariate skewness and kurtosis [58]. The results showed that skewness = 1.13, *p* < 0.001, and kurtosis = 30.84, *p* < 0.001. Although these results failed to meet the conventional cut-off for multivariate skewness and kurtosis (skewness < ±1; kurtosis < ±20) [58], we did not transform the data in the subsequent analysis [59]. This was done based on the sufficient sample size of this study and our dependent variable being normally distributed (see Table 1). Moreover, to identify possible multivariate outliers, we calculated the minimum covariance determinant estimator of the Mahalanobis distance using SPSS; a chi-square probability of 0.001 was regarded as the criteria to exclude the cases [60]. In this study, nine cases were detected as outliers, which were omitted from the subsequent analysis. In addition, a preliminary investigation revealed less than 1% missing values per each study variable and, thus, the full information maximum likelihood estimation was used to handle missing data in the linear regression [61].

Furthermore, as illustrated by prior research [25,35], many researchers have reported a low-to-moderate association between the two dimensions of grit. Likewise, we aimed to incorporate targeted two- and three-way interactions among study variables in the linear regression. Given these empirical indications and our research objectives, we performed two separate linear regression models, one for each dimension of grit. This was done to potentially decrease the multicollinearity issues, which may bias the variance of the coefficient estimates and make the estimates very sensitive to minor changes in the model [62,63]. Nevertheless, given the conceptual linkage between two dimensions of grit [25], we controlled another aspect when regarding one of the targeted dimensions as the predictor.

In terms of possible significant interactions, we performed simple slope analysis using R software package Jtools [64] to probe the nature of the interactions [65]. Although simple slope analysis is widely used to probe interactions, such an approach has been criticized, as identifying representative values of the moderator is subject to arbitrary guidelines for determining the values [66]. To compensate for this limitation, we also employed the Johnson–Neyman technique, which identifies regions of the significance of moderator values [67]. In these analyses, the significance level was interpreted at *p* < 0.05, and the 95% confidence intervals did not contain zero.

## 3. Results

### 3.1. Preliminary Analysis

Means, standardized deviations, skewness and kurtosis values, and zero-order correlations are presented in Table 1. As shown in Table 1, the values of skewness and kurtosis indicated univariate normality of the study variables. In terms of correlations, teacher autonomy support and perseverance were moderately and positively related to social competence, whereas consistency was weakly and positively associated with social competence.

### 3.2. Linear Regression Analysis

The results of when perseverance is regarded as the independent variable, and consistency is treated as a covariate are shown in Table 2; the model explained the 27.7% variance of social competence. To be specific, teacher autonomy support and perseverance were each positively linked to social competence. With regard to the interaction terms, the results only exhibited a significant two-way interaction between perseverance and students’ gender. Notably, to create the three-way interaction term, we should establish lower levels of interaction terms (i.e., two-way interaction) sequentially [57,68], although the interaction between the two moderators (i.e., two dimensions of grit and gender) was not our research focus. Therefore, in this study, we did not further interpret this two-way interaction term. In terms of covariates, students from families with higher SES reported higher social competence levels than those from lower SES.

The results of when consistency is regarded as the independent variable, and perseverance is treated as a covariate are shown in Table 3; the model explained a 28.0% variance of social competence. Specifically, teacher autonomy support was positively related to social competence. In terms of covariates, family SES and perseverance were each positively linked to social competence. Moreover, two-way interaction between teacher autonomy support and consistency was positively associated with social competence; three-way interaction among teacher autonomy support, consistency, and students’ gender was also positively related to social competence. In addition, it should be noted that in the case of significant three-way interaction, the interpretation of a single main effect and two-way interaction is basically incomplete or misleading [65,69]. Statistically, obtaining a significant direct effect of the independent variable or moderators on social competence is not the prerequisite for further interpreting significant interaction terms in the linear regression model [65]. For example, in many empirical studies [38,70], the authors have reported significant interactions with nonsignificant main effects (e.g., a crossover interaction). Therefore, in this study, we only interpreted significant three-way interaction without further commenting on significant two-way interaction in this section.

**Figure 2:**
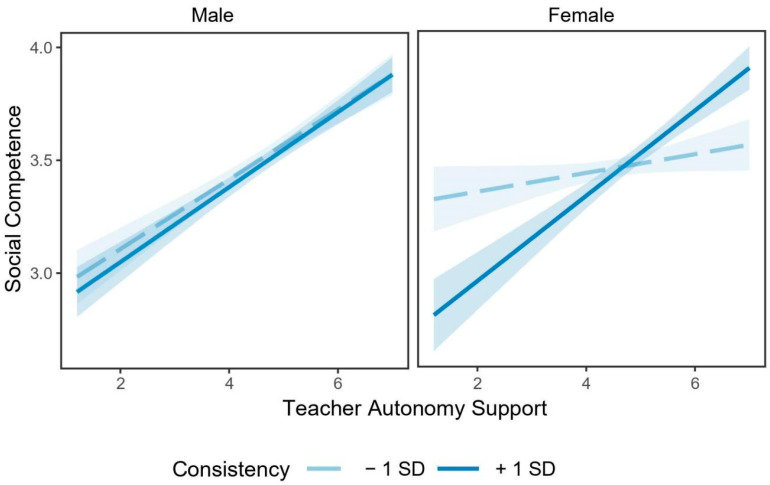
Interaction effect of teacher autonomy support, consistency, and students’ gender on social competence. Note: *N* = 1009. Consistency was divided into two levels based on mean: low = *Mean* − 1 *SD*, high = *Mean* + 1 *SD*.

The examination of the pattern of the three-way interaction with simple slope analysis revealed that when reporting higher levels of consistency, the association between teacher autonomy support and social competence was significantly positive for both males (*b* = 0.16, *SE* = 0.02, *t* = 7.13, *p* < 0.001) and females (*b* = 0.18, *SE* = 0.03, *t* = 5.80, *p* < 0.001). By contrast, when reporting lower levels of consistency, this association was still significant for males (*b* = 0.15, *SE* = 0.02, *t* = 5.90, *p* < 0.001), but not for females (*b* = 0.04, *SE* = 0.03, *t* = 1.25, *p* = 0.21; see Figure 2).

**Figure 3:**
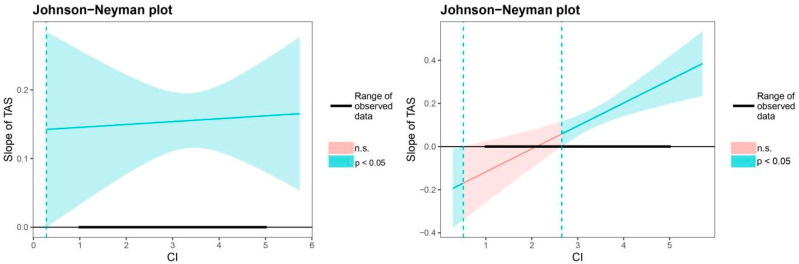
Johnson–Neyman regions of significance and confidence bands for the conditional relationship between teacher autonomy support and social competence as a function of consistency of interests, separately for males (**left** side) and females (**right** side). Note: *N* = 1009; TAS = teacher autonomy support; CI = consistency of interests; n.s. = non-significant.

Analyses of this three-way interaction with the Johnson–Neyman technique identified that when consistency was inside the interval ranging from 0.51 to 2.65, the slope of teacher autonomy support was not significant for female students (*p* < 0.05; see Figure 3).

## 4. Discussion

Although previous research has documented the beneficial effect of teacher autonomy support on individuals’ adaptive psychosocial functions, such as social competence [20,22], this association is still less clear-cut among undergraduate students in a collective culture context. Moreover, the role of individual characteristics in this linkage has not been comprehensively addressed in the existing literature. Attempting to fill these research gaps, this study, following the socioecological framework, investigates the association between teacher autonomy support and social competence and further examines the moderating role of two facets of grit (i.e., perseverance and consistency) and gender in this association among Chinese undergraduate students. The result showed that teacher autonomy support was positively associated with social competence. Moreover, when reporting higher levels of consistency, this association was significantly positive for both males and females; by contrast, when reporting lower levels of consistency, this association was only significant for males but not for females.

The first purpose of this study is to investigate the association between teacher autonomy support and social competence of Chinese undergraduate students. In line with our first hypothesis, the results showed that teacher autonomy support was positively associated with social competence. This finding further confirms SDT [16,17] and extends emerging literature documenting the beneficial role of autonomy support on Chinese individuals’ optimal functioning [14,71,72]. One possible explanation is that with rapid economic development and the ever-increasing cultural exchanges between Chinese society and Western societies, independence and autonomy in Chinese culture are more emphasized than before [72]. This may be particularly highlighted for undergraduate students, as during this period, many developmental transitions occur. For example, undergraduate students begin to seek job opportunities, leave their parents’ homes, and get involved in intimate relationships [73]. These changes, linked to novel experiences and social roles, make undergraduate students more autonomous from their family system [73,74]. Likewise, undergraduate students spend most of their time in college, and the role of teacher and student becomes one of the significant relationships influencing their social values and behaviors. By validating students’ perspectives, teachers may give students a sense of control and mastery [22], which further facilitates students’ initiation of relationships and other related social skills.

The second purpose of this study is to investigate the moderating role of grit and students’ gender in the association between teacher autonomy support and social competence. Different from our second hypothesis, we found that consistency moderated this association, after controlling for perseverance. By contrast, perseverance did not exhibit a significant moderating role in the association between teacher autonomy support and social competence, after controlling for consistency. One possible explanation is that perseverance and determination are highly valued among Chinese individuals [28,75]. In this context, perseverance is universally underscored in terms of the social adjustment of students, which may be independent of contextual influences such as teacher autonomy support. Another interpretation is in line with prior research [38], illustrating that the significant moderating role of perseverance is highlighted in terms of “at-risk” populations, such as migrant youth. It is noteworthy that the current sample is a group of “typical” undergraduate students and, thus, the moderating role of perseverance may not be so pivotal. Moreover, it should be noted that the existing literature of grit has mostly focused on academic outcomes, highlighting the salient role of perseverance instead of consistency [30,31]. In the current study, distinctive from academic outcomes, we centered on social competence, and the interactive role of the two dimensions of grit could be different. Due to the scarcity of literature documenting this issue, further research should extend or replicate the current study in other populations and cultural contexts to understand whether there is a conceptual gap of the different roles of perseverance and consistency on psychosocial outcomes. Additionally, as suggested by prior research [76], the findings of the moderated multiple regression can be restrained by the study sample and possible methodological artifacts (e.g., self-report questionnaires), which may alter the magnitude of the moderating effect. Therefore, this finding should be interpreted with those limitations in mind.

Concerning significant three-way interactions, the results showed that when reporting higher levels of consistency, the association between teacher autonomy support and social competence was significantly positive for both males and females. By contrast, when reporting lower levels of consistency, this positive association was significant only for males but not females. In other words, for male students, the positive association between teacher autonomy support and social competence is independent of the levels of consistency; for female students, this positive association is highlighted in higher (but not lower) levels of consistency. One possible explanation is that autonomy-supportive teachers can significantly influence Chinese male students’ social behaviors, as they do not usually keep a “close” relationship with teachers. Due to gender norms, males are expected to be more independent and prone to be emotionally less expressive than females [43,77]. In this context, salient support from significant figures, such as teachers, can have a heightened effect on male students’ social competence, which is independent of the levels of consistency. In terms of female students, high autonomy support from teachers and great consistency jointly optimize their social competence. As documented by prior research [78], females are emotionally sensitive to others’ needs and interactions, which may, to some degree, influence the stability of social relationships. In this perspective, high consistency, emphasizing the commitment to long-term orientations and goals, as well as contextual support from teachers, may jointly help female students establish harmonious and stable interactions.

To our knowledge, this is the first empirical study documenting the process of the association between teacher autonomy support and social competence under the cultural background of collectivism. To briefly summarize, the current study contributes to the existing literature in the following manner. First, we further enrich the beneficial role of autonomy support in undergraduate students’ social competence in a collective cultural context. Second, we explore the interactive role of grit on social adjustment, which goes beyond academic outcomes. Moreover, we unpack two dimensions of grit and demonstrate that great consistency is crucial to the development of social competence, particularly for female students. Third, the current study is built on a large and ethnically diverse sample of Chinese undergraduate students, which increases our confidence with respect to interpreting these findings. This is because the extant literature of Chinese individuals has mainly focused on the ethnic majority populations (the Han ethnic group), which decreases the representativeness of the research samples.

### Limitations

Along with these significant findings, the current study may involve several limitations that should be acknowledged here. First, the cross-sectional design employed in this study cannot infer the causality of study variables. For instance, students with a high capacity for social skills may report high levels of teacher autonomy support. Future research is highly recommended to conduct a longitudinal design to examine the reciprocal or reverse associations between these study variables. Second, the current study solely relies on self-report measurements, which may inflate the study associations. This is because empirical study building on self-report questionnaires is potentially influenced by social desirability and common method bias [79]. Although social desirability is partially controlled, as we highlighted some points (e.g., please answer as honestly as possible, and there are no right or wrong answers in this survey) in the instructions, we cannot entirely exclude this bias due to the inherent methodological weakness. In addition, the current study assesses the perception of teacher autonomy support, but not actual teacher autonomy support. Therefore, it is suggested that future studies should obtain external informants (e.g., teachers) to ascertain these study associations further. Third, although teacher autonomy support has been demonstrated as one of the significant influences on undergraduate students’ adaptive functioning [71,72], this study fails to consider other salient sources of autonomy support, such as peers. This is because undergraduate students spend an extensive amount of time with peers, who are regarded as another critical figure of emotional and social support [80]. Fourth, certain characteristics of our sample limit the generalizability of the research findings. For example, participants were recruited based on a convenience sampling method, and thus the current findings may be, to some degree, impacted by volunteer bias. That is, students who volunteer to participate may be distinctive from those who choose not to. In this regard, the current sample may not be representative of some inherent and unobservable attributes. It is highly recommended for future initiatives to use probability sampling methods to increase the representativeness of the sample. In addition, students in this study are solely recruited from one province of mainland China. Considering the regional differences in China, recruiting a nationally representative sample is valuable. Moreover, focusing only on Chinese undergraduate students delimits the generalizability of findings to other cultural groups. Future research should consider including study samples from different cultural contexts to provide a comprehensive understanding of the cross-cultural applicability of the study associations.

## 5. Conclusions

Although traditional Chinese culture emphasizes the teacher–student relationship hierarchy and authority, our findings support the critical role of teacher autonomy support in undergraduate students’ social competence. Moreover, this study provides solid evidence of the process of this expected linkage, which may lay a fine-grained foundation for university-based intervention or prevention programs. Apart from enhancing autonomy support from teachers, individual characteristics, such as consistency, should also be incorporated into the programs, particularly for female students.

## Figures and Tables

**Table 1:** Descriptive statistics and bivariate correlations of study variables for Chinese undergraduate students.

Variable	*M*	*SD*	Range	Skewness	Kurtosis	1	2	3	4	5	6	7
1. TAS	4.64	1.04	1–7	−0.21	0.36	-						
2. Perseverance	3.40	0.77	1–5	0.08	−0.24	0.29 ***	-					
3. Consistency	3.28	0.72	1–5	−0.17	0.48	0.15 ***	0.23 ***	-				
4. Gender ^a^	-	-	1–2	-	-	0.06 *	−0.08 **	−0.05	-			
5. Social Competence	3.48	0.55	1–5	0.09	0.45	0.38 ***	0.44 ***	0.11 ***	−0.02	-		
6. Age	20.66	1.30	18–25	-	-	−0.10 ***	0.06 *	0.04	−0.04	−0.04	-	
7. Socioeconomic Status	0.00	3.76	−4.99–16.39	-	-	0.03	−0.07 *	0.02	0.10 ***	0.05	−0.18 ***	-

Note: *N* = 1009; ^a^ coded as 1 = male, 2 = female; TAS = teacher autonomy support. * *p* < 0.05, ** *p* < 0.01, *** *p* < 0.001.

**Table 2:** Linear regression analysis predicting social competence from teacher autonomy support, perseverance, and students’ gender.

Variables	*b*	*b SE*	95% CI		*t*	*p*
TAS	0.14	0.02	0.11	0.17	9.25	<0.001
PE	0.25	0.02	0.21	0.29	12.12	<0.001
Gender ^a^	−0.03	0.03	−0.09	0.03	−0.99	0.32
Age	−0.01	0.01	−0.03	0.01	−0.77	0.44
SES	0.01	0.01	0.01	0.02	2.73	0.01
CI	−0.01	0.02	−0.05	0.03	−0.39	0.70
TAS × PE	−0.02	0.02	−0.05	0.02	−0.92	0.36
TAS × Gender	−0.02	0.03	−0.09	0.04	−0.81	0.42
PE × Gender	−0.09	0.04	−0.17	−0.01	−2.10	0.04
TAS × PE × Gender	0.01	0.04	−0.06	0.08	0.22	0.82

Note: *N* = 1009; ^a^ coded as 1 = male, 2 = female; TAS = teacher autonomy support; PE = perseverance of effort; CI = consistency of interests; SES = socioeconomic status.

**Table 3:** Linear regression analysis predicting social competence from teacher autonomy support, consistency, and students’ gender.

Variables	*b*	*b SE*	95% CI		*t*	*p*
TAS	0.14	0.02	0.11	0.17	8.92	<0.001
CI	−0.01	0.02	−0.05	0.03	−0.53	0.59
Gender ^a^	−0.04	0.03	−0.10	0.02	−1.42	0.16
Age	−0.01	0.01	−0.03	0.01	−0.83	0.41
SES	0.01	0.01	0.01	0.02	2.58	0.01
PE	0.26	0.02	0.22	0.30	12.55	<0.001
TAS × CI	0.05	0.02	0.02	0.09	2.83	0.01
TAS × Gender	−0.05	0.03	−0.10	0.01	−1.53	0.13
CI × Gender	0.01	0.04	−0.07	0.10	0.34	0.74
TAS × CI × Gender	0.09	0.04	0.02	0.17	2.44	0.02

Note: *N* = 1009; ^a^ coded as 1 = male, 2 = female; TAS = teacher autonomy support; CI = consistency of interests; PE = perseverance of effort; SES = socioeconomic status.
